# Masculine guardianship in family systems: decoding cinematic discourses of protection, abuse and institutional failure in *Chithha* movie

**DOI:** 10.3389/fsoc.2026.1871284

**Published:** 2026-08-28

**Authors:** R. Krishika, S. Saravanan

**Affiliations:** 1 VIT School of Design, Vellore Institute of Technology, Vellore, India; 2 VIT School of Media, Arts and Technology, Vellore Institute of Technology, Vellore, India

**Keywords:** Bronfenbrenner systems, child sexual abuse, family system, sociological film analysis, Tamil cinema

## Abstract

**Introduction:**

The Tamil film *Chithha* (2023) addresses the growing relevance of child sexual abuse in native cinema contexts. The study examines how narratives about care, trust and abuse create and disrupt the portrayal of masculine guardian roles.

**Methods:**

Using critical discourse analysis, key scenes were selected and interpreted through Bronfenbrenner's ecological systems theory to connect individual experiences of harm with broader family, community, and institutional structures.

**Results:**

The narrative explores multiple layers of theoretical approaches for analyzing child sexual abuse with intricate relationships between the environmental factors that contribute to a child's development, fostering more complex and equitable narratives within the cinematic realm.

**Conclusion:**

The findings demonstrate that child sexual abuse is presented in *Chithha* as an ecological and familial transgression shaped by the perpetrator's proximity, kinship trust, gendered silence and institutional constraints within interconnected social systems. A key finding is the demonstration of secondary victimization in the exosystem through procedural delays, community scrutiny and fractured support mechanisms that compound the child's trauma, extending beyond the abusive event itself.

**Policy Implication:**

The study contributes to family sociology by showing how cinematic discourse can function as a cultural site for understanding the intersections of gender, caregiving, and family violence with implications for public awareness and policy conversations related to child safety, gender equality, and social justice.

## 1 Introduction

In developing nations like India, tackling the urgent issue of child sexual abuse (CSA) is not just necessary, it's a groundbreaking endeavor that demands our collective attention and action, with studies indicating that over 50% of children have experienced some form of sexual abuse. The high prevalence is often attributed to various sociocultural and systemic factors, including power imbalances, lack of awareness, and inadequate protective measures (Choudhry et al., 2018). This signifies an issue with the lack of clarity regarding the definition of CSA. The diverse terminology used by professionals, such as child molestation, sexual victimization, and child sexual assault, which complicates the understanding of the problem. So Browne and Finkelhor (1986) suggest that a 5 year age difference should be considered for defining abuse in children under 12, and a 10 year difference for adolescents aged 13-16, emphasizing the importance of context in definitions. Childhood experiences play a pivotal role in shaping an individual's psychological wellbeing in adulthood. Among these experiences, childhood maltreatment, which encompasses psychological, physical, and sexual abuse, has profound and often lasting impacts. The interdependencies of these different forms of maltreatment are crucial for understanding their combined effects on mental health outcomes. A study by Briere Runtz and Runtz (1987) indicates that multiple types of abuse often occur within the same families, suggesting that a comprehensive assessment of all forms of maltreatment is necessary to understand their overlapping effects on psychological symptoms later in life. Bagley and Ramsay (1986) and Peters (1998) suggest that family dynamics, including maternal warmth and parental support, are critical in predicting psychological difficulties in adulthood. Low maternal warmth was identified as a strong predictor of later psychological issues, indicating that neglectful family relations may significantly contribute to the outcomes of CSA (Fry, 1993). A study quantifying the prevalence of CSA shows considerable rates among both boys and girls. For instance, 1:11 boys and 1:3 or 1:4 girls had experienced sexual abuse during childhood or adolescence (Whitla, 1991). Within family sociology, it is emphasized that familial bonds, which enable caregiving, also create an environment where abusive behavior turns into common practice.

The literature indicates a troubling trend where the number of cases is rising, despite the enactment of the Protection of Children from Sexual Offenses Act^1^ (POCSO Act, 2012), which aimed to impose stricter penalties on offenders. Human Rights Watch report (2013): titled “Breaking the Silence: CSA in India,” indicates that it is significantly less reported compared to sexual violence against women. It emphasizes that over 50,000 children are abused annually, as report integrated regionally, with many cases going unreported, highlighting the gravity of the situation. World Vision India study (2014-15): a comprehensive survey involving 45,844 respondents across 26 states found that one in every two children is a victim of sexual abuse. Alarmingly, 90% of children under 10 years old are deemed unsafe, which stresses the urgent need for protective measures. National Crime Records Bureau (2016): This report documented 1,06,958 cases of crimes against children in 2015-16, with 36,022 classified as sexual offenses. It also noted that social stigma often prevents families from reporting abuse, adding to the complexities. Government report on child care institutions (2017): This report revealed that out of 9,000 child care institutions in India, nearly 4,000 are unregistered, making children in these facilities particularly vulnerable to abuse. The report prompted a social audit to investigate the conditions of these institutions (Nair, 2019). The National Crime Records Bureau reported a 4.5% increase in crimes against children from 2018 to 2019, resulting in 1,48,185 incidents in 2019 of these, 31.2% were under the POCSO Act (2012). Maharashtra had the highest number of POCSO cases, with 8,503 from 2017 to 2019, highlighting serious concerns about child protection and legal enforcement challenges. Based on data obtained from the National Crime Records Bureau, there were a total of 47,221 cases reported under the POCSO Act (2012) in the year 2020. The most recent data published by the National Crime Records Bureau indicates that there were 1,49,404 reported cases of crimes against children in 2021. Notably, 53,874 of these cases, representing 36.05 percent, were classified under the POCSO (Tyagi and Karande, 2021). The evidence shows that systemic failures keep making victims vulnerable throughout the years of case studies which have been conducted. Family sociology explains that domestic violence and child maltreatment exist because social problems emerge from the power dynamics which society assigns to different genders and the breakdown of family structures. The cultural texts of cinema function as a research center because they show family dynamics while they educate viewers about different methods of caregiving, guardianship, and protection.

### 1.1 Intersection of Cinema

The CSA in Indian cinema, especially Kollywood (refers to the Tamil-language film industry that is based in Chennai, India) (Selvaraj, 2008; Velayutham, 2008). It has often faced criticism for its portrayal of gender-based violence, particularly against women. Many films depict scenes of domestic violence, sexual assault, and emotional abuse, reinforcing harmful stereotypes and perpetuating a culture of violence against women. Female characters in South Indian cinema are portrayed from a male gaze perspective (Christensen, 2018). These portrayals often trivialize or romanticize abuse failing to recognize the power imbalance between the perpetrator and the victim, where films frequently portray sexual violence through a patriarchal lens, films like *Nisabdham* (2017), *Ratsasan* (2018), *Paavakadhaigal* (2020), *Netrikann* (2021), *Gargi* (2022), *Sembi* (2022) and series like *Suzhal: The Vortex* (2022) illuminate the harsh realities of physical and sexual abuse of children and women, portraying the struggles faced by victims and sparking important conversations about these social issues and emphasizing themes such as family honor and masculinity while neglecting the trauma experienced by victims (Karupiah, 2023) whether the victim is a woman or a child. This portrayal aligns with alarming statistics in India, where over 53% of children face sexual abuse, predominantly by individuals known to them (Kumar et al., 2012). This also demonstrates that “war-as-honor beliefs” are enacted through the physical bodies of women and queer individuals who are considered outsiders to the male-centric system (Väyrynen et al., 2021).

However, not all South Indian films contribute to a negative depiction; instances exist where movies have treated these issues with sensitivity, providing a platform for dialogue and change. Studies around sexual abuse on viewer perceptions indicate that Malaysian Indian audiences of Tamil cinema frequently struggle to distinguish between consensual pre-marital sex and non-consensual sexual violence, which indicates that the cinematic language itself creates confusion about these important ethical and legal boundaries (Karupiah et al., 2022). The CSA cases depend on two essential elements, which include power imbalance and the child's inability to give legal consent. The absence of clear and direct representation allows societal myths to continue, which downplay the seriousness of CSA cases while presenting on screen as they wrongly assign guilt to the victim, a pattern that public health research on gender-based violence has established as common. These aspects of CSA are evident in actual data from India, which cinematic stories often fail to convey. The statistics show that girls become victims at higher rates than boys, but boys experience extensive sexual assault, which people tend to overlook because of social stigma and toxic masculinity that links victim status to weakness and the false belief that “boys cannot be raped” (Sharma, 2022; Swathisha and Deb, 2022). As a result, this perspective is rarely represented on screen.

Scholars have increasingly considered child sexual abuse through ecological, trauma-informed, and sociocultural lenses, highlighting the interplay of family relationships, institutional responses, and broader cultural norms (Oral et al., 2016; Wiehe, 2021). Research on masculinity and family sociology has also challenged conventional notions of male guardianship, kinship trust and home authority. Yet, there has been little attention given to the visual representation of these interlinked discourses in Tamil cinema through cinematic language. But the research establishes that cinema functions as a space which connects “cinematic projections” with “everydayness, virtual circulations, musical ramifications and political assertions” to achieve a full understanding of masculinity (Chakraborty, n.d.). For example, the film *Chithha* (Arun Kumar, 2023) was inspired by the novel “Bitter Chocolate” (Pinki, 2000) addresses sexual violence against children in the native cinema^2^ (Pandian, 1996), providing an alternative lens of existing masculinity and presenting how visuals can expose power dynamics and gender constructs within the domestic space, shedding light on the challenges women face in their everyday lives. By showcasing women's mundane^3^ (Smith, 1992) yet oppressive experiences. Cinema urges the audience to question and challenge patriarchal norms, which are actively working to break away from traditional stereotypes and redefine the portrayal of women by showcasing their strength, resilience, and agency. In doing so, cinema plays a crucial role in shaping and influencing the audience's mindset and society at large toward greater gender equity. The significance of this study lies in understanding the representation of CSA in the film *Chithha* as a form of a childcare movie^4^ (Willett, 2008). While *Chithha* has garnered scholarly attention for its depiction of CSA, gender issues, and social justice, it can provide insights into societal attitudes, the effectiveness of media in raising awareness, and potential avenues for intervention and prevention strategies. The film used emotional elements together with psychological elements, investigative elements and legal elements to present its main subject matter about child abuse and harassment (Karthiga et al., 2014). Few studies have investigated how film language influences the understanding of CSA within interconnected social contexts using a sociological approach. To address this gap, this study combines Fairclough (2023) critical discourse analysis (CDA) with Bronfenbrenner et al. (1998) ecological systems theory (EST). It examines how film language, familial relationships, institutional practices, and cultural concepts collaborate to construct meanings of CSA. By situating the film within the realms of family sociology and masculinity studies, this research expands the existing scholarship. It illustrates how abuse is created, concealed, and reinforced through interactions across various social systems.

EST provide a powerful framework that is used to study how human behavior develops through different social environments (Bronfenbrenner et al., 1998). Which is most widely used in studies of violence and abuse because they enable them to understand CSA as a result of multiple factors, which include family weaknesses and institutional shortcomings, community silence and socio-cultural norms. Heise (1998) argues that multiple levels of causation should be used to define violence because individual history, relationship patterns, community contexts, and societal structures all contribute to violent behavior. The approach of this framework switches the analysis from focusing on “bad individuals” to examining “enabling systems” within organizations.

The ecological systems model is not often referenced in cinema, especially in Tamil cinema, but it is often implicitly reflected in narratives focusing on child development themes. A prime example is the Tamil film *Pasanga-2* (2015), which delves into the complexities of child care and serves as a vehicle to examine this ecological model of development. However, Ecological models serve as effective tools in CSA research because they demonstrate how both perpetrators and institutional and cultural factors enable abuse through systems that fail to protect children, law enforcement that operates poorly, and societal attitudes that blame victims of sexual violence (Finkelhor, 1994b; Heise, 1998). These models demonstrate that disclosure functions as a social process that people negotiate based on their feelings of fear and trust and their understanding of power dynamics (Herman, 1992). Ecological frameworks enable film's depiction of both the relationship between victims and their abusers and the social conditions that make people stay silent about their experiences.

Thus, EST acts as the main analytical lens for this study to understand how *Chithha* illustrates CSA across many entangled social systems. Unlike traditional modes of thinking concerning abuse as a part of a troubling social (or interpersonal) event, it considers child development within abuse as the interconnected, nested social structures of the microsystem, macrosystem, exosystem, mesosystem, and chronosystem. Through the lens of this theory, the study is concerned with how latent and active constraints shape/influence the concealment, the revealing and the aftermath of CSA. By marrying EST with CDA, the study is concerned with what the film communicates in textual form and the way the film structures the silences, the absences, the institutional and social framing, and the responsibility of social accountability. Future Tamil cinema must shift away from sensationalism to create better public knowledge and prevention strategies through its research-based and ethical approach, which applies trauma-informed care principles while treating CSA as a serious health emergency that affects all genders and not just as a storytelling device for retributive justice plots.

## 2 Methodology

In line with the study's objectives, the film *Chithha* (2023) is scrutinized and analyzed, as cinema functions as both a cultural representation and an ideological discourse. The study uses the CDA and EST frameworks to examine the portrayal of CSA in the film across levels and layers. The cinematic techniques and the discursive practices level explore how these elements shape meanings around trust, masculinity, and trauma; and the social practice level interprets these discourses through EST. The eight scenes were purposively sampled because they are the most discursive moments in *Chithha* where CSA is explicitly or symbolically constituted through dialogue, character interaction, visual composition and institutional engagement. Selection was based on two criteria. First, substantial narrative discourse concerning research objectives and CDA is linked in each scene. Second, the scenes chosen represent each of Bronfenbrenner's five proposed ecological systems: familial interactions (microsystem), community relationships (mesosystem), institutional responses (exosystem), sociocultural ideologies (macrosystem), and the chronosystem, reflecting the changing impact of trauma over time. Thus, the chosen scenes represent a comprehensive view of the film's ecological discourse, while maintaining the analytical depth necessary for a qualitative analysis of cinema. The study also adheres to specific inclusion and exclusion criteria, particularly utilizing discourse analysis. This approach provides a comprehensive framework for examining the complex narratives of female characters, focusing on their experiences of abuse and the nuanced aspects of discrimination. The analysis incorporates EST to trace findings from micro to macro levels of systemic violence. As the film *Chithha* serves as a distinctive case study, depicting CSA ethically and illustrating how families, communities, and institutions collaborate to address disclosures, trauma, and issues of justice. Additionally, it explores child development through the intricate interactions of various environmental contexts, all nested within each other, ranging from the immediate family to broader cultural influences. This theory is specifically applied to the child protagonist and female protagonist in the film, rendering this approach particularly suitable for analyzing the mirrored character development.

### 2.1 Unit of analysis

**Figure 1:**
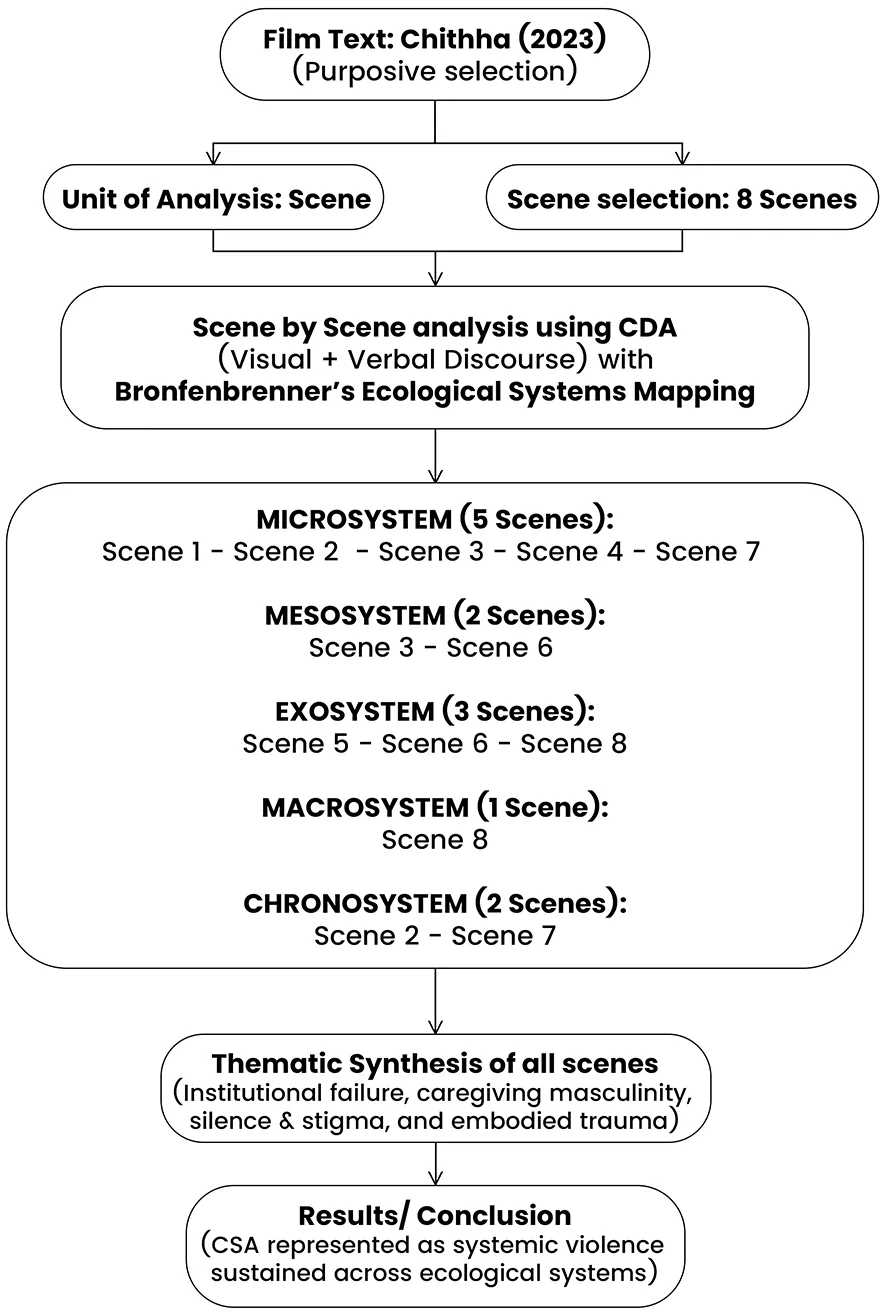
Represents the integrated EST research flow design.

The film's “scenes” serve as the primary research unit since it exists as a continuous part of storytelling that maintains a unified environment, time period, and action sequence. A scene reaches its final state when the story introduces a new location or time period or a different focus of the plot. As illustrated in Figure 1, the scene-based research method works effectively because it allows researchers to divide the film into smaller parts, which show how characters speak, how they interact and how visual elements and symbolic content support the research objectives of the study. Each scene functioned as a complete audiovisual component, which included both spoken and silent communication and showed ideological viewpoints and depictions of gender and institutional authority. The interpretation of these elements relies on CDA, which connects them to EST.

### 2.2 Inclusion criteria

Scenes were included if they contained at least one of the following:

- The use of explicit or implicit language about CSA, which included references to abuse, suspicion, grooming, fear or aftermath.
- The display of child trauma, together with physical vulnerability and mental withdrawal.
- The existence of silent denial alongside shame and stigma resulting in victim-blaming.
- The representation of masculine caregiving, together with parental duties, which men share.
- The existence of institutional engagement through police response, community monitoring activities, and legal processes.

### 2.3 Exclusion criteria

Scenes were excluded if they contained any of the following:

- The film used cinematic elements which did not relate to CSA discourse since they included ordinary statutory warnings and production-style inserts.
- The content was duplicated, yet no fresh discursive content was introduced.
- The content presented information about the cast and production without showing any essential parts of the story.

The study selected 8 key scenes, which it used to analyse the film through five different levels according to Bronfenbrenner's ecological systems model.

## 3 Findings

The CDA framework enables researchers to study how language and representation create lasting power dynamics and social hierarchies, and ideological dominance. In cinema, discourse goes beyond spoken dialogue to include all non-verbal elements such as silences, metaphors, gaze and visual symbolic components. The study also integrates EST, which shows how human experiences result from multiple environmental interactions. With it, the researchers used the ecological model to create their operational system through the following methods:

**Microsystem:** immediate relationships (child, caregiver, family, perpetrator proximity).

**Mesosystem:** interactions between immediate settings (family-school, family-community).

**Exosystem:** indirect institutional structures affecting the child (police response, legal processes, community networks).

**Macrosystem:** cultural ideologies, patriarchal norms, stigma, gendered moral regulation.

**Chronosystem:** time-based development (trauma progression, delayed justice, evolving awareness).

### 3.1 Narrative framework of *Sundari*

*Sundari*^5^ (played by Sahasra Sree) is a pivotal character in the film *Chithha* (Arun Kumar, 2023), representing a nuanced portrayal of a young girl approximately 10 years old. Her character is depicted with complexity, illustrating the imperfections inherent in childhood. One salient narrative thread involves *Sundari* succumbing to peer pressure, exemplified by her act of stealing, which serves as a lens through which the film explores the societal pressures faced by children in educational settings. A scene particularly illustrates *Sundari's* desire for a colored chicken, which her uncle *Eeswaran* (played by Siddharth) initially refuses to purchase, citing her inevitable mortality and her potential regret. This exchange escalates into a challenge where *Eeswaran* prompts her to calculate the outstanding balance owed to the grocery shopkeeper for the chicken. The outcome, where she receives a lollipop instead, highlights her innocence and exposes her vulnerability, a thematic focus of the film that seeks to protect and advocate for childhood innocence. *Sundari* embodies a typical child archetype, playful, reliant on adult guidance, and largely oblivious to the more insidious dimensions of societal complexities. Her relationship with *Eeswaran* serves as the narrative's emotional core, reflecting themes of familial love and the intricacies of custodial responsibilities. The narrative plot of her disappearance is a critical event that not only propels the story forward but also serves as a commentary on child abuse and the phenomenon of missing children. This aspect of her character addresses these serious societal issues while avoiding sensationalism, instead fostering a conversation about community attitudes toward child safety and collective societal responsibility. Ultimately, *Sundari* symbolizes hope and the imperative to safeguard children's innocence amid societal challenges.

### 3.2 *Chithha* through the lens of CDA and EST

The study has cultivated a deeper understanding of the intricate social and cultural risk factors associated with child maltreatment and the various social adjustment challenges that may arise (Williams, 2022). The disintegration of family environments emerges as a critical factor highlighted by Bronfenbrenner et al. (1998), which can lead to a cascade of negative outcomes for youth. These outcomes may include feelings of alienation and apathy, as well as behaviors such as rebellion, delinquency, and even violence (Lang, 2020). According to Bronfenbrenner's theory, an individual develops through interactions with different environmental systems, which function together over time (Hertler et al., 2018). The film studies framework enables researchers to develop detailed narratives that illustrate how CSA affects individuals and their connections to broader social systems that shape their lives. Child abuse manifests in various forms, including intimate partner violence, and is situated within a broader context that profoundly impacts developmental trajectories within this ecological model. The way that these diverse forms of abuse interact with environmental factors emphasizes the complexity of the issue at hand (Flynn and Mathias, 2023). This analysis aims to reveal the various roles that different characters play in shaping the experiences of children and to delineate how these relationships serve to protect or endanger children from sexual predators. Each layer of the model will be illustrated with a specific scene from the film, underscoring its influence and involvement in the children's lives. Table 1-**Scene 1** represents the initial layer and the life of the victim before being abused. She is seen in a caring environment with everyday innocence. Table 1 presents the selected scenes from the film with the framework that helped to explain why family dysfunction creates conditions amenable to CSA, as family members are typically the first to detect and report abuse (Hassan et al., 2015). The 57% of children who did not resist showed this behavior because their attackers belonged to their familiar social circles, which they trusted. The ecological system framework shows that offenders operate from the child's microsystem, which consists of family members, caregivers and all other individuals who maintain continuous contact with the child, which creates situations that lead to potential abuse (Martinello, 2020). The dangerous situation develops because offenders use their close relationship with victims to create circumstances where they can abuse their victims without detection. Public spaces become unsafe because of alcohol-related public disturbances and gang activities, prostitution, graffiti vandalism and the existence of rundown buildings (Holman and Stokols, 1994). These serve as locations for CSA and make it difficult to reveal abuse because of fear and shame (McGill and McElvaney, 2023). The research shows that situational factors which lead to abuse occur in locations where children can access restricted areas, and there are inadequate personal, social and legal protections, and there is insufficient monitoring (Wortley, 2018). The first noted risk element for sexual victimization occurs when people come into contact with isolated places, which specifically include those areas where sexually driven offenders operate. Pezzoli et al. (2020) demonstrates that offenders specifically select victims who display certain characteristics, which include intellectual disabilities and externalizing problems. Safe environments need active monitoring systems together with distinct protective regulations and adult education about grooming patterns (Powell, 2007).

**Table 1:** Presents the selected scenes from the film *Chithha* (2023) along with CDA and EST.

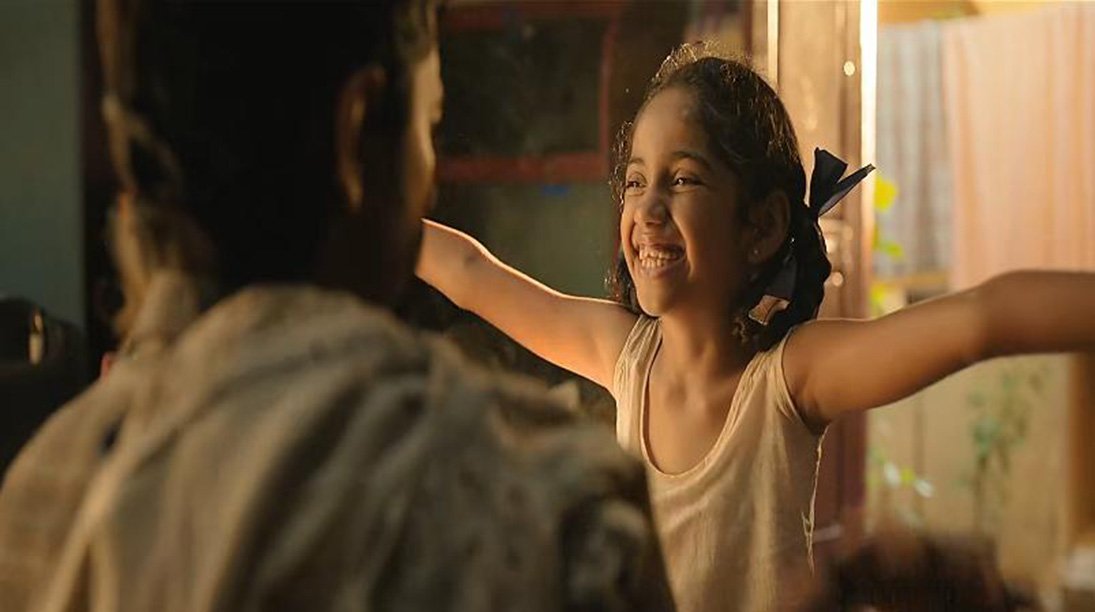 **Scene 1 represents the everyday innocence of the child protagonist**.	
**Narrative context**	The child protagonist is in a caring environment that emphasises stability and routine. This establishes emotional attachment between the child and the caregiver.
**Key visual evidence**	Sundari is framed in mid-close-up shot with her uncle, utilising warm lighting and eye-level angles to create a sense of intimacy and security.
**Key discourse evidence**	Everyday dialogue and playful communication reinforce safety.
**CDA interpretation**	The film constructs childhood safety as socially produced through family care. This later becomes a critique because the safety collapses due to systemic failure.
**Ecological mapping**	Microsystem.
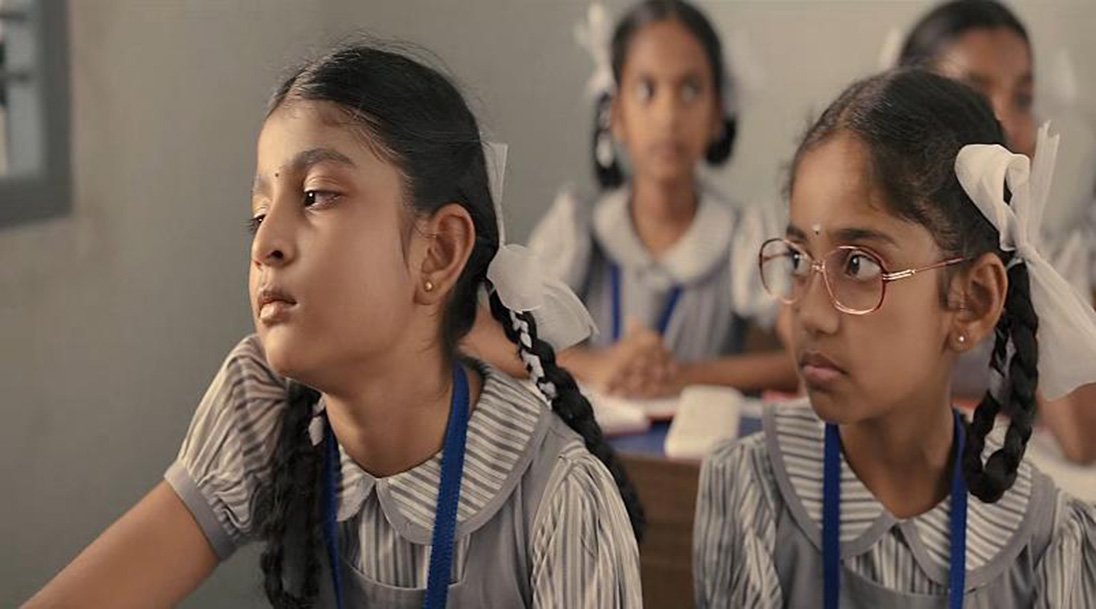 **Scene 2 represents a scene of the first victim (the child protagonist's friend) and her post-abuse silence**.	
**Narrative context**	The child protagonist's friend becomes a victim of CSA, establishing that abuse existed before the main protagonist's incident. Her post-abuse behaviour changes demonstrate her trauma through her patterns of fear, withdrawal and avoidance.
**Key visual evidence**	The film doesn't explicitly show; rather utilizes mid-close-up shots to significantly capture Sundari's worried expression as she interacts, while her distressed friend underscores the trauma of the first victim.
**Key discourse evidence**	Silence becomes dominant. There is minimal dialogue, suggesting trauma is not easily narratable and her reduced speech, avoidance of eye contact, and hesitation to engage with adults.
**CDA interpretation**	The lack of explicit speech highlights how CSA remains a taboo topic in our culture. This silence acts as a form of discourse, illustrating how abuse is concealed and the child is denied a voice. Trauma often finds expression through non-verbal communication. In this context, the child's body becomes a canvas for inscribed violence, revealing the deep scars left by such experiences.
**Ecological mapping**	Microsystem + chronosystem (direct abuse within intimate proximity with immediate impact, leaving trauma over time).
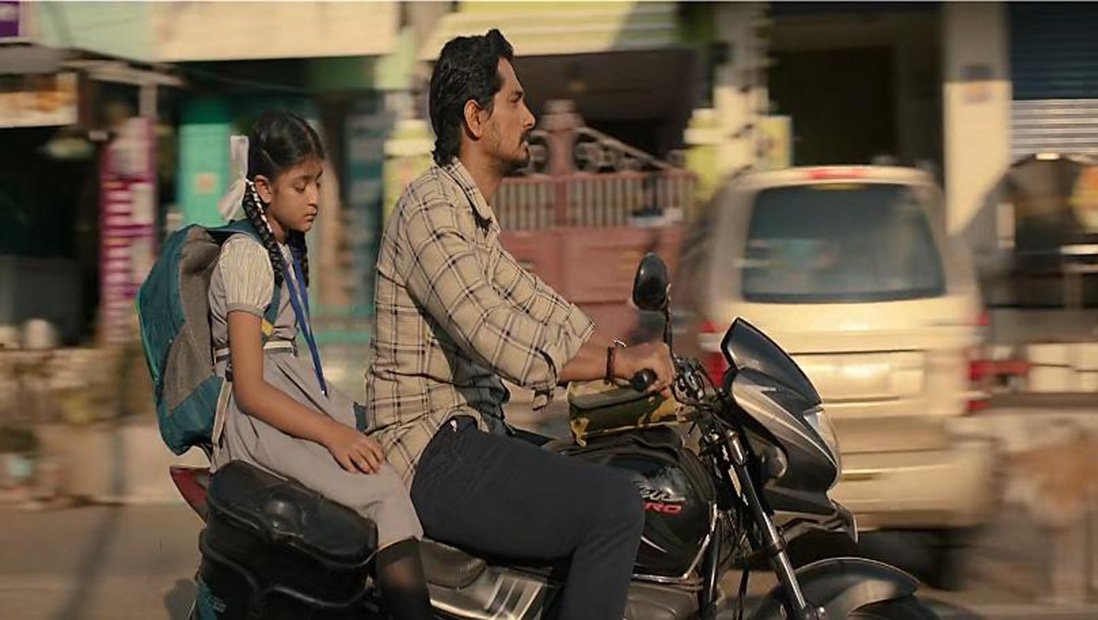 **Scene 3 represents an adult unaware of the signs.**.	
**Narrative context**	The child shows early behavioural warning signs (fear, withdrawal, discomfort), but adults dismiss them as normal or temporary, avoiding serious concern.
**Key visual evidence**	The scene is a wide shot of the child and adult on a motorcycle, and there is a spatial gap that indicates emotional and psychological distance. The child is isolated and distressed; the adult is oblivious, constructing a discourse of invisible suffering and unrecognised trauma.
**Key discourse evidence**	Adults use minimising language (“nothing serious,” “don't overthink”) and avoid directly naming abuse. Silence becomes a key communicative element.
**CDA interpretation**	Denial functions as a discursive strategy that protects adult comfort and social reputation, while the child's voice and bodily cues are later realised.
**Ecological mapping**	Microsystem + mesosystem.
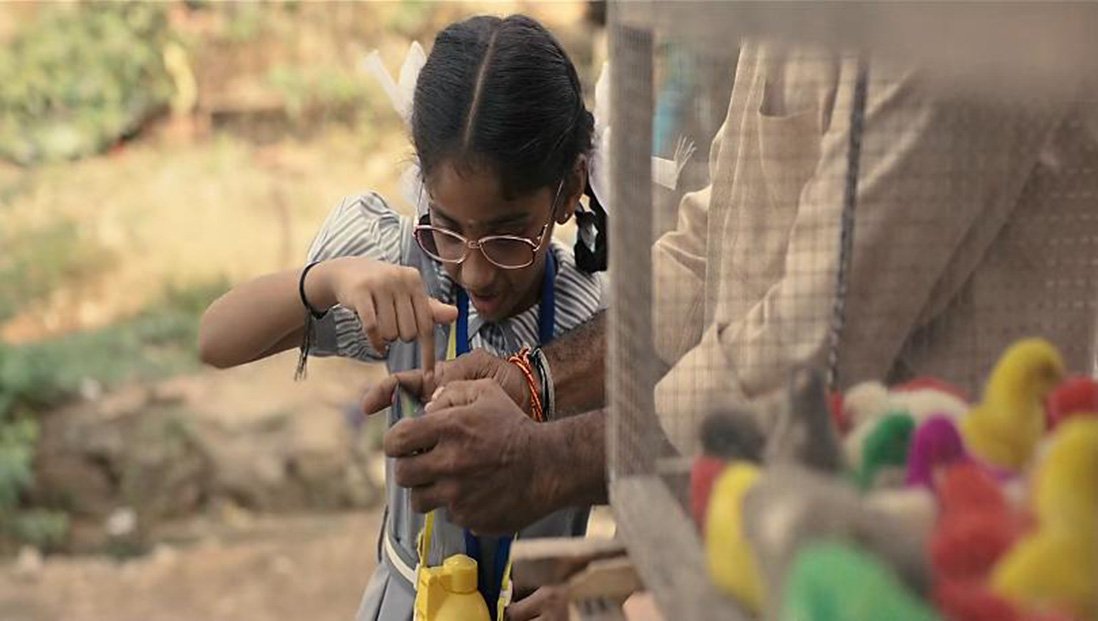 **Scene 4 illustrates the perpetrator's opportunity to access the main victim**.	
**Narrative context**	The perpetrator's opportunity emerges through routine movements and social invisibility. The film suggests how abuse is enabled by ordinary environments.
**Key visual evidence**	High-angle shot of Sundari, expressing enthusiasm upon seeing her favourites: colored chicken and a mobile game highlighting her innocence, while the perpetrator's face is obscured to enhance the scene's intensity.
**Key discourse evidence**	Transitional spaces such as streets or semi-private locations show how children can be taken without immediate suspicion. There may be minimal dialogue; the danger is implied through visual tension.
**CDA interpretation**	The scene shows how perpetrators use social normalcy to remain invisible. Power operates through invisibility and silence.
**Ecological mapping**	Microsystem.
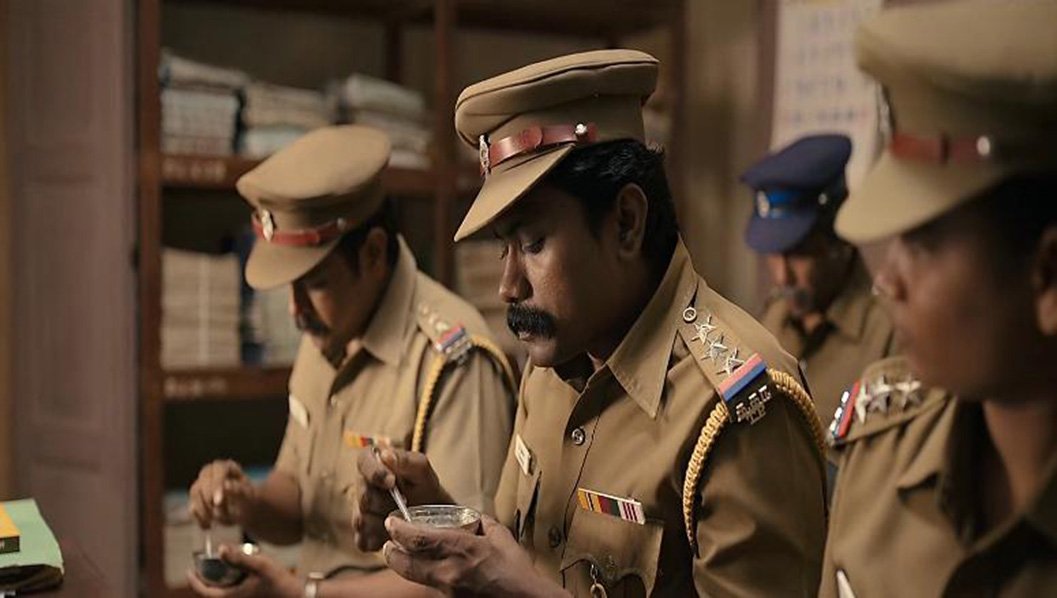 **Scene 5 represents the systemic neglect**.	
**Narrative context**	The caregiver approaches the police, but institutional procedures delay action.
**Key visual evidence**	A wide shot of the systemic protectors sharing a brief meal, despite the seriousness of the missing child situation, reflects their struggle with responsibility and the challenges they face in balancing care with urgency.
**Key discourse evidence**	Procedural language dominates questions, forms, waiting, and scepticism.
**CDA interpretation**	The institutional discourse constructs the victim's family as petitioners rather than urgent survivors. The state is represented as a gatekeeper of justice.
**Ecological mapping**	Exosystem.
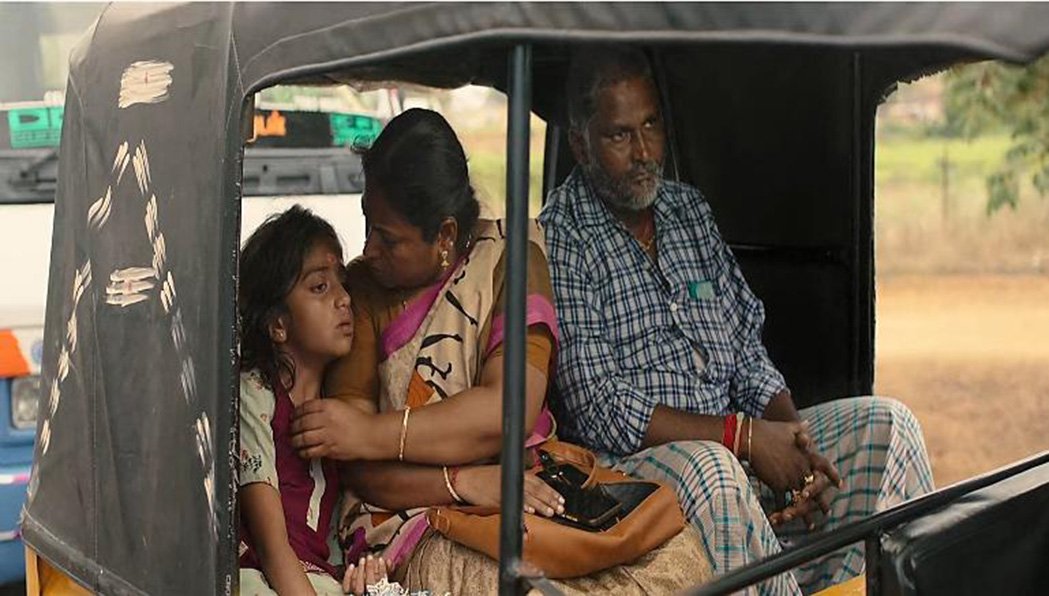 **Scene 6 represents the child rescued (Protection Narrative)**.	
**Narrative context**	The child is found through the intervention of a random person or outsider. She is found on public transport.
**Key visual evidence**	A medium wide shot of public transport shows a lady in shock at the child's distress, emphasising Sundari's vulnerability.
**Key discourse evidence**	The framing often contrasts feelings of helplessness with assistance; the rescuer is portrayed as a moral agent. Supportive language, reassurance, and protective gestures are evident.
**CDA interpretation**	The scene constructs rescue as an ethical counter-discourse, showing that care can emerge outside institutions.
**Ecological mapping**	Exosystem + Mesosystem (community-level intervention).
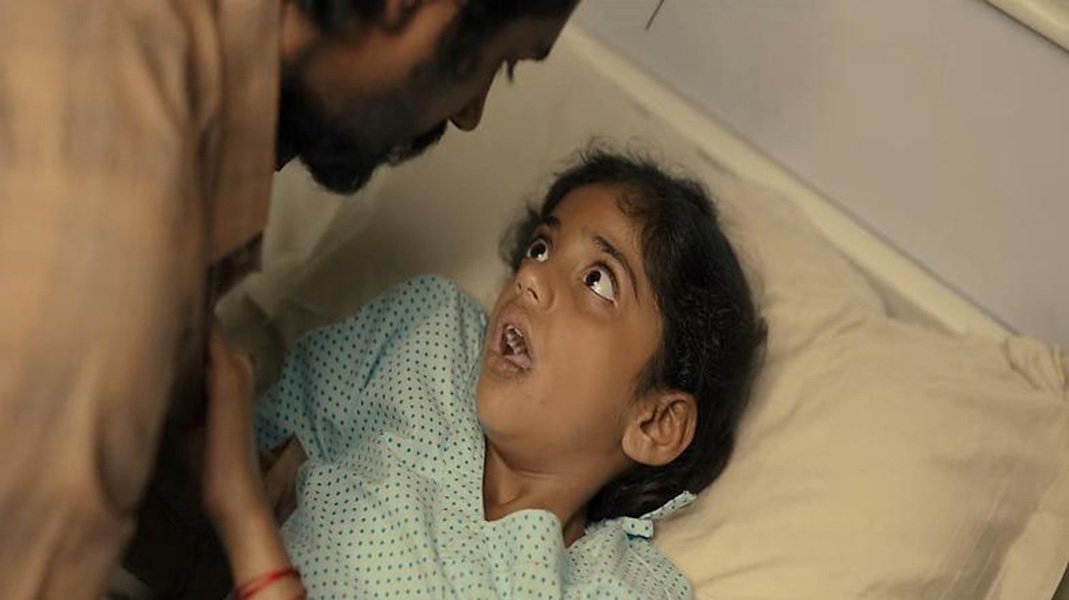 **Scene 7 illustrates the embodied fear after the trauma**.	
**Narrative context**	After returning, the child's trauma becomes visible through withdrawal, fear, and silence.
**Key visual evidence**	Close-up shot of Sundari, with a fearful expression, emphasises her trauma. Once, she felt close and safe with her uncle, but after the trauma, she struggles to process it around anyone.
**Key discourse evidence**	Silence dominates; the child does not articulate pain easily.
**CDA interpretation**	Silence is not absence but a form of communication shaped by trauma and stigma. The child's body becomes the site of memory around adults, as shown in Scene-8.
**Ecological mapping**	Microsystem + chronosystem.
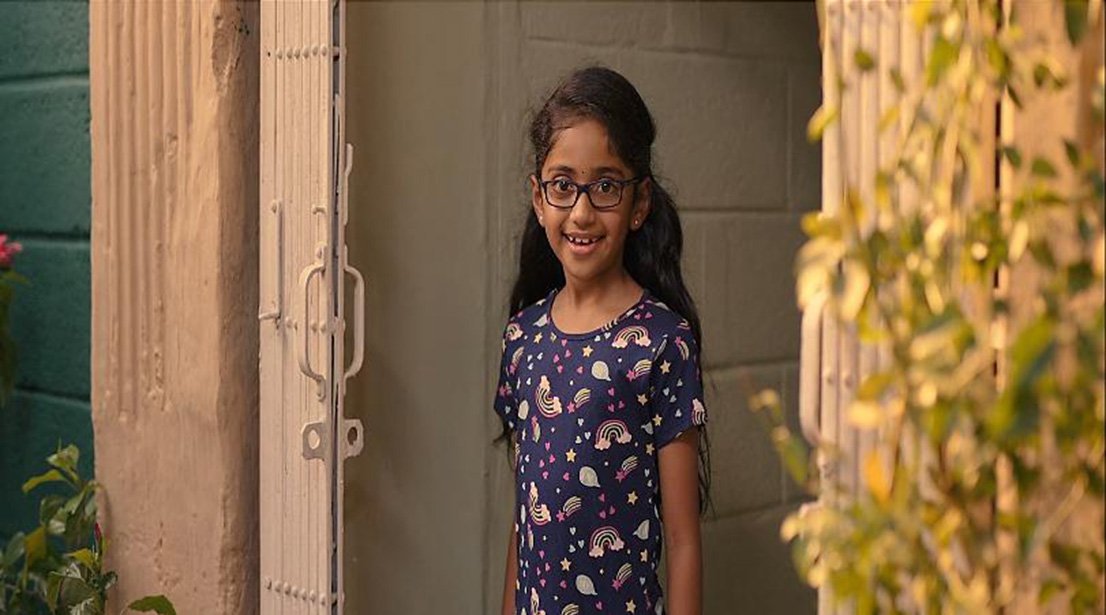 **Scene 8 illustrates the healing and return to normal life**.	
**Narrative context**	The film ends with the child gradually returning to routine life, indicating recovery.
**Key visual evidence**	Brighter framing, open spaces, and positive body language suggest emotional restoration.
**Key discourse evidence**	Supportive family speech, normal dialogue returning.
**CDA interpretation**	The film constructs healing as possible but dependent on supportive relationships. The ending frames resilience as a counter-narrative to violence.
**Ecological mapping**	Exosystem + Macrosystem (recovery over time shaped by cultural meaning).

The study results demonstrate that *Chithha* (2023) shows CSA as a pervasive social problem, which people maintain through their refusal to discuss it, their social stigmatization, and their inability to speak about it. The film narrates how abuse operates in common spaces and becomes part of typical social behavior. Through its selected scenes, it demonstrates that family bonds and daily routines do not protect children from danger, as the multiple ecological systems determine their safety. The temporal context of CSA can be viewed through three major phases of abusive experiences, which include the pre-abuse stage, the period of CSA abuse until its end as the abuse phase and the time after the abuse ended as the post-abuse phase. The child's social and physical surroundings determine the development of all three CSA stages (Holman and Stokols, 1994). Victims, on the other hand, frequently infringe on relationships between acquaintances and friends, demonstrating a large group of pedestrian abuses. One theory regarding male perpetrators of CSA is that their physical strength enables them to control child victims, which they use to fulfill their attachment needs and intimacy desires that normal relationships do not satisfy (Hassan et al., 2015).

CSA develops from trusted domestic spaces (microsystem), rather than from unfamiliar external threats. The friend of the child protagonist, who had experienced earlier victimization, shows how attackers obtain access to their victims through relationships and close contact. In this case, the perpetrator was the auto driver who took the victim to an isolated area. He was familiar to the victim's family, school, and the local community, which is why the child felt safe approaching him. The film avoids explicit depiction and instead uses symbolic framing, closed spaces, restricted movement, and fearful facial expressions to indicate violence. Close-ups and mid-shots emphasize the child's emotional distress as presented in Table 1-**Scene 2**, establishing that the victim's post-abuse behavior changes illustrate her trauma through patterns of fear, withdrawal, and avoidance. The CDA process shows that people treat this particular act as standard behavior, which occurs between people and kids when adults direct kids to follow their orders and to stay silent. The child displays warning signals through their withdrawal and discomfort. As illustrated in Table 1-**Scene 3**, the child appears distressed and fearful about what has occurred, while the adult remains unaware of the situation. This denial becomes a significant finding because it shows that the earliest enabling condition of CSA is not the abuse act itself but the adult's refusal to interpret childhood distress as a serious indicator of harm.

The next victim of CSA is the child protagonist, who experiences abuse when the perpetrator uses his social invisibility to enter her microsystem and watches her routine movements until he finds an opportunity to isolate her. The perpetrator offers her the colored chickens, which she likes and mobile games, as shown in Table 1-**Scene 4**, to create a distraction which he uses to take her away from her safe area into an unmonitored space where he will complete his abusive plan. The friend's earlier abuse strengthens the systemic argument by proving repetition and pattern, which transforms the protagonist's case from an isolated tragedy into a broader critique of social structures.

The study demonstrates at its mesosystem level how family and community ties create social reactions toward CSA. The neighborhood joins the search because of collective panic, but the film uses this moment to criticize both surveillance and gossiping practices. The CDA analysis shows that community conversations develop into judgmental attitudes, which force families to endure public scrutiny instead of receiving compassionate support. The study reveals that people in the community react in two ways because they want to help with rescue efforts, but their actions create stigma, which prevents people from talking about their problems. Through depicting mesosystemic and macrosystemic factors, films can explore the complexity of disclosure pathways, including social stigma, institutional betrayal, and cultural silence (McPherson et al., 2025). It also demonstrates how CSA acts as a social control mechanism which uses shame and reputation management to create social boundaries within tightly bound social groups.

The exosystem findings show that violence has an institutional aspect which needs to be studied. The police complaint and investigation scenes show how the police system creates obstacles through its slow procedures, which establishes distance from its operations. The police station operates under the assumption that no significant problems exist because officers show their solidarity by sharing and consuming sweets within the station with no worries, as shown in Table 1-**Scene 5**. The film presents this delay as secondary victimization because the institutional aspect extends trauma duration while increasing psychological suffering. Bronfenbrenner's model strengthens this reading by showing how systems outside the child's immediate life can still decisively shape survival outcomes and justice processes. On the other side, a random woman shows concern, doubts the perpetrator, and saves the child by informing the legal authority. As shown in Table 1-**Scene 6**, the woman appears as a rescuer and a moral agent for the child protagonist, who was abused and tortured.

The macrosystem examines cultural beliefs which shape societal attitudes and legal frameworks that define the environment for CSA. The ecological model shows how cultural elements, which include patriarchy, sexual objectification, stigmatization, and abuse normalization, create barriers that prevent CSA survivors from being seen and heard (Martinello, 2020; Tao, 2025). The macrosystem level of Chithha demonstrates how patriarchal belief systems continue to keep CSA abuse cases hidden. The film demonstrates how honor culture, together with social stigma, causes adults to avoid stating their experiences of sexual violence. The use of silence serves as a cultural practice which maintains social stability instead of protecting children.

The chronosystem enables films to show how social awareness about CSA has evolved throughout history and how abuse affects survivors and their journey from recovery to posttraumatic growth. The study examines changes in family relationships and social protection policies and how people handle their stress throughout their life, while also considering the historical time periods that shape CSA reporting and handling (Hartley et al., 2016; McGill and McElvaney, 2023). The chronosystem findings show that ongoing psychological damage follows rescue operations because the child shows post-return withdrawal and silence, as shown in Table 1-**Scene 7**, which indicates permanent psychological damage and where the child cannot articulate the pain easily. The process of healing unfolds through gradual steps, which need ongoing caregiving, plus emotional security and a period of time for recovery. The experience of abuse leads to “place aversion” because victims develop permanent reminders of their trauma through their interaction with specific environmental elements. The process results in people abandoning their main personal spaces, which include their bedrooms and homes that serve as safe spaces for them to feel at home. The required approach to tackle CSA requires examination of both personal and family elements, together with the environmental factors that either enable or stop such abuse from happening (Holman and Stokols, 1994). The study discovered that children face increased abuse risk because of their daily activities and their social interactions with others beyond their home environment. The study found that over 50 percent of non-familial abuse incidents happened within a distance of 2.5 kilometers from where the offenders lived (Chopin and Caneppele, 2019). The findings showed that environmental factors raise abuse risk, yet every environment remains vulnerable to child sexual abuse (Finkelhor, 1994a).

These Environments or circumstances are where the child is isolated, unsupervised, or vulnerable to manipulation. Perpetrators often take advantage when children are physically alone or emotionally abandoned from their usual protective networks, a “safer circle.” Locations that are private and lack adult supervision, such as certain parts of the home or non-institutional places, pose higher risks. Molesters may also exploit risky online environments where children lack appropriate awareness or supervision (Chasan-Taber and Tabachnick, 1999; Patterson et al., 2022). The distance between offenders and their victims determines which methods should be used to stop their crimes. The existing programs, which currently focus on educating children and protecting victimized groups, need to shift their focus according to emerging frameworks, which require them to protect people who are at risk of becoming offenders and who have direct contact with children before any abusive behavior takes place (Knack et al., 2019; Smallbone et al., 2008).

The findings go beyond simply describing the narrative. They look at how cinematic discourse shapes the meanings of CSA through visual language and character relationships. Instead of showing abuse as a separate criminal act, the film connects masculinity, familial trust, institutional authority, and cultural silence as related social processes. Techniques like framing, lighting, spatial composition, camera movement, and symbolic mise-en-scène strengthen these ideas by placing CSA within larger ecological structures that influence vulnerability, disclosure, institutional response, and long-term recovery. Thus, each scene is seen as a space where individual experiences interact with broader family, community, institutional, and cultural systems. The narrative reflects a shifting societal consciousness toward CSA, indicative of the evolving awareness within Indian cinema and society. It showcases a growing willingness to confront these issues head-on, paving the way for dialogue and education. While the film does not explicitly depict the intergenerational impacts of abuse, it strongly suggests the long-term psychological effects on victims. It underscores the urgency of breaking cycles of violence and silence that can persist through generations. A clean and protective environment is essential for a child's growth. *Chithha* offers an ethically grounded portrayal of CSA in Tamil cinema by showing trauma alongside caregiving and systemic accountability.

### 3.3 Mirrored character development

The film *Chithha* presents a significant character arc of how a victim gradually overcomes, but the trauma remains. This narrative is explored through two different stages (victim stage, survivor stage), represented by two characters. The parallel stories of Sundari, the child victim, and Sakthi, the adult survivor, play a key role in the discussion of CSA in modern Tamil cinema. They shift the focus from punishing offenders to supporting survivors and addressing long-term trauma. The study investigates the primary character development of *Sundari* and *Sakthi*, who play central roles throughout the story. As the narrative unfolds, the mirrored arc development allows for a gradual evolution and growth of the character, culminating in a significant resolution or metamorphosis by the conclusion. This evolution is typically shaped by a series of experiential encounters and confrontations with obstacles that promote a deeper comprehension of the character's journey and its impact on the overall narrative structure. A meticulously crafted character arc is paramount in effective storytelling, adding layers of depth and complexity that allow for audience engagement on a more profound emotional plane.

The character arcs of young *Sundari* and adult *Sakthi* present a compelling exploration of their intertwined lives, illustrating how their experiences reflect a mirrored phase of existence where their roles are effectively reversed. Both characters endure significant trauma from childhood abuse, a shared experience that profoundly shapes their individual struggles and ultimately impacts their long-term emotional and psychological wellbeing as adults. This shared trauma serves as a crucial element in understanding how they navigate the myriad challenges that life presents. One of the key distinctions between the two characters lies in the way they each manage their trauma. For *Sakthi*, the adult survivor, the experience of sexual trauma is marked by isolation. In her moment of need, she finds herself without support, grappling with the aftermath alone. This solitary battle emphasizes a crucial aspect of trauma recovery: the importance of having a supportive network. The absence of familial or emotional support in *Sakthi's* life illustrates how such isolation can prolong the healing process, making recovery all the more difficult.

In stark contrast, *Sundari* the young victim, experiences a significant shift. During a similar traumatic incident, adult Sakthi steps in from the sidelines, actively advocating for *Sundari* by encouraging *Eeswaran* to be present and supportive. This intervention signifies a pivotal change in their narrative and serves as a form of empowerment for *Sundari*. *Sakthi's* efforts to foster a supportive environment during *Sundari's* ordeal not only aid in *Sundari's* healing journey but also play a crucial role in mitigating the potential psychological scars that such trauma can leave behind. The concept of the chronosystem enhances the narrative by allowing the film to explore the evolution of social awareness surrounding CSA. It sheds light on how societal perceptions have shifted over time, highlighting the long-lasting effects of abuse on survivors. It maps the journey of these characters from their traumatic experiences through the complex pathways of recovery, eventually leading into the realm of posttraumatic growth. This intricate portrayal not only emphasizes the characters interpersonal dynamics but also invites viewers to engage with the broader societal implications of trauma and healing, ultimately fostering a deeper understanding of the issues surrounding CSA and the critical nature of support in recovery processes. Through this mirrored narrative, *Chithha* shows that abuse is not just a single incident but continues to affect identity, memory, trust, and social belonging throughout life. This links childhood victimization with adult survival as part of a lasting trauma journey.

The study examines changes in family relationships and social protection policies and how people handle their stress throughout their life while also considering the historical time periods that shape CSA reporting and handling (Hartley et al., 2016; McGill and McElvaney, 2023). In the future, *Sundari* may evolve into an independent, self-made woman like *Sakthi*. The intensity of events unfolding in *Chithha* adds layers to the character's complexity. The director invites viewers to pause at the river of cinema, encouraging a moment of reflection on the journey of these characters before they continue, momentarily setting aside their own packed and demanding lives. This visual enigma skillfully encapsulates the enigmatic nature of their past experiences. The film challenges the common Tamil cinematic theme of retributive justice, which usually resolves trauma through revenge or punishing the abuser. Instead, it suggests a more complex approach to healing that relies on emotional support, protective caregiving, responsiveness from institutions, and accountability from the community. This comparative narrative broadens the understanding of justice beyond legal remedies to include recognition, support, and healing. This makes *Chithha* an important work for understanding how cinema can recast child sexual abuse as a social and intergenerational issue rather than just a criminal act.

## 4 Conclusion

This study investigates the Tamil film *Chithha* (2023), which serves as a sociological text to explore the portrayal of family, masculine guardianship, and systemic violence in CSA. The analysis employs CDA alongside Bronfenbrenner's EST. The film conceptualizes CSA not merely as an isolated act of individual deviance but as a form of violence perpetuated through interconnected family, community, and institutional structures. Through CDA, the study reveals that the film's dialogue and visual presentation depict silence as a communicative method that maintains social order, yet fails to protect children. The film's chronology highlights the earlier victimization, which serves as a pivotal narrative moment that transforms the protagonist's case into a broader system, characterized by community silence and institutional processing delays. Utilizing the EST framework, the analysis traces the story's development, demonstrating that the film focuses on the microsystem, where children endure their most intense traumatic experiences through physical contact and emotional detachment, compounded by their caregiver's mental health challenges, which create stigma at the mesosystem level. The Exosystem scenes illustrate that institutional responses, through policing and procedural mechanisms, result in secondary victimization due to their sluggish and unresponsive approaches. The macrosystem establishes a social framework that perpetuates patriarchal systems and honor-based traditions, fostering tendencies toward silence and evading responsibility. The chronosystem reveals how trauma persists throughout life, influencing an individual's emotional stability and identity development. The study's discourse highlights the institutional system's delayed response to abuse, highlighting how formal structures often mirror the cultural denial present within the family environment. Overall, the study emerges as a significant Tamil cinematic text that diverges from sensational portrayals of sexual violence, offering a socially grounded critique of CSA as a multi-layered socio-cultural crisis. This further contributes to gender and film scholarship by demonstrating how the integration of CDA with ecological theory provides a robust methodological approach for analyzing CSA representation in cinema. However, the study is limited by its focus on a single film and does not include audience reception data. Future research should extend this approach to comparative Tamil and Indian films and incorporate audience studies to evaluate how such cinematic representations influence public understanding, awareness, and discourse on CSA. The study underscores a global concern for child welfare, gender equality, and justice, illustrating that sociologists can gain a deeper understanding of domestic violence through the study of films.

## Data Availability

The original contributions presented in the study are included in the article/supplementary material, further inquiries can be directed to the corresponding author.

## Footnotes

1 The Protection of Children from Sexual Offences Act, 2012, is key legislation in India aimed at preventing child sexual abuse and exploitation. It creates a legal framework to protect minors, defines sexual offences, imposes strict penalties on offenders, and establishes special courts for swift justice, all while promoting a child-friendly judicial process.

2 Native cinema produces films which Indigenous and local communities create to show their actual life experiences, cultural identity, language and worldview. The Native Cinema creates resistance through its films, which show Indigenous and local community life.

3 The term “mundane women” describes research which examines the daily existence of common women by studying their nonexceptional activities, their daily household work and their typical ways of living.

4 In research terms, a child care movie is a cinematic text that represents childhood as a vulnerable social category and explores how family, community, and institutions shape a child's safety, development, and well-being.

5 A minor, specifically a girl under the age of 18, has been selected to portray the character Sundari. The director detailed the communication process with Sahasra's parents, emphasising that sensitive information was withheld to protect her. It was also noted that she had a positive and enjoyable experience during her time on set.
