# Correction: Masculine guardianship in family systems: decoding cinematic discourses of protection, abuse and institutional failure in *Chithha* movie

**DOI:** 10.3389/fsoc.2026.1986464

**Published:** 2026-09-11

**Authors:** R. Krishika, S. Saravanan

**Affiliations:** 1 VIT School of Design, Vellore Institute of Technology, Vellore, India; 2 VIT School of Media, Arts and Technology, Vellore Institute of Technology, Vellore, India

**Keywords:** Bronfenbrenner systems, child sexual abuse, family system, sociological film analysis, Tamil cinema

Affiliation [^1^VIT School of Design, Vellore Institute of Technology, Vellore, India. ^2^VIT School of Media, Arts and Technology, Vellore Institute of Technology, Vellore, India] was omitted from the paper. This affiliation has now been added for author(s) [R. Krishika^1^, S. Saravanan^2^].

The contact email address for the corresponding author, S. Saravanan, was erroneously given as *saranrulz671@gmail.com*. The correct corresponding email address is [s.saravanan@vit.ac.in].

The original version of this article has been updated.

